# Genome-Wide Identification of the RR Gene Family and Its Expression Analysis in Response to TDZ Induction in *Rhododendron delavayi*

**DOI:** 10.3390/plants12183250

**Published:** 2023-09-13

**Authors:** Lvchun Peng, Xuejiao Li, Yan Gao, Weijia Xie, Lu Zhang, Jie Song, Shifeng Li, Zhengxiong Zhao

**Affiliations:** 1 College of Agriculture and Biotechnology, Yunnan Agricultural University, Kunming 650201, China; green315@126.com; 2 Flower Research Institute of Yunnan Academy of Agricultural Sciences, National Engineering Research Center for Ornamental Horticulture, Kunming 650205, China; 3 College of Landscape and Horticulture, Yunnan Agricultural University, Kunming 650201, China; 4 College of Resources and Environment, Yunnan Agricultural University, Kunming 650201, China; gy2360450910@163.com

**Keywords:** *Rhododendron delavayi*, adventitious bud regeneration, RR gene family, segmental replication

## Abstract

The cytokinin response regulator (RR) gene is essential for cytokinin signal transduction, which plays a crucial role in plant growth and development. Here, we applied bioinformatics to *Rhododendron delavayi*’s genome to identify its RR gene family and systematically analyzed their gene characteristics, phylogenetic evolution, chromosomal localization, collinearity analysis, promoter cis-elements, and expression patterns. Overall, 33 *RdRR* genes were distinguished and classified into three types. All these genes harbored motif 5 (YEVTTVNSGLEALELLRENKB), the most conserved one, along with the plant-conserved domain (REC domain), and could be mapped to 10 chromosomes with four gene pairs of segmental replication events but no tandem replication events; 13 *RdRR* genes showed collinearity with *Arabidopsis thaliana* genes. Promoter analysis revealed multiple hormone-related *cis*-elements in the RR genes. After a TDZ (thidiazuron) treatment, 13 genes had higher expression levels than the control, whose magnitude of change depended on the developmental stage of leaves’ adventitious buds. The expression levels of *RdRR14*, *RdRR17*, *RdRR20*, and *RdRR24* agreed with the average number of adventitious buds post-TDZ treatment. We speculate that these four genes could figure prominently in bud regeneration from *R. delavayi* leaves in vitro. This study provides detailed knowledge of *RdRRs* for research on cytokinin signaling and *RdRR* functioning in *R. delavayi*.

## 1. Introduction

Cytokinins are important phytohormones involved in the regulation of plant growth and development, leaf senescence, nutrient uptake, source–sink interactions, and plant responses to biotic and abiotic stresses [1]. Many studies have found that cytokinins promote cell division [2,3,4], plant organ development [5,6,7], and responses to environmental stimuli [8,9,10], all of which are pertinent to plant morphogenesis and crop yields. Cytokinins function critically in the cytokinin signal transduction system, which consists of three parts: the receptor protein histidine kinase, the phosphate group transfer protein, and the response regulator (RR) [11,12]. The cytokinin RR is a key factor in cytokinin signaling and has the most family members of cytokinin-related regulatory genes [13,14]. These regulate downstream signals by modulating transcriptional and protein activity, and they have the potential to coordinate many physiological processes that are cytokinin-based.

*Rhododendron delavayi* is an evergreen shrub or small tree that belongs to the *Hymenanthes* subgenus in the Ericaceae family. This woody plant is widely distributed, exhibiting strong drought tolerance; its flowers are large, red-colored, and have great ornamental value [15]. These traits make this species a key material for *Rhododendron* breeding. *Rhododendron* plants are mainly used for ornamental purposes, the greening of slopes, flower cutting, and vase planting, to name a few. However, due to seasonal and extreme weather impacts, it is generally difficult for *Rhododendron* seeds to take root under natural environmental conditions, limiting the rapid propagation and marketization of plants in this genus. Therefore, it is necessary to find ways to achieve rapid propagation in a short time.

Tissue culturing can overcome the limitations imposed by natural conditions on the growth and development of *Rhododendron*, accelerate its breeding programs, and help achieve its large-scale, commercial production in a shorter time. Efficient differentiation of adventitious buds from in vitro leaves is a pivotal mode of plant regeneration. Thidiazuron (TDZ) can significantly promote the differentiation of adventitious buds from in vitro leaves of *R. delavayi*, and type-A *Arabidopsis* response regulator genes (*ARR6*, *ARR9*, *ARR17*) were upregulated compared to treatment without TDZ during adventitious bud formation [16]. However, the differentiation ability of differing varieties of *Rhododendron* varies greatly when induced by TDZ. The RR gene is known to be a key factor in cytokinin signal transduction, a process critically involved in bud differentiation [17]. In the present study, the *RdRR* gene was first identified and then used to distinguish candidate genes for cytokinin-induced bud formation, followed by the systematic analysis of their physicochemical properties, gene structure, chromosome distribution, evolutionary characteristics, and expression patterns. This work lays a foundation for future research on gene cloning and function in *Rhododendron* and provides an important basis for related research on promoting the plant regeneration and development of *R.* *delavayi* in particular.

## 2. Results

### 2.1. Phylogenetic Tree Analysis of R. delavayi RR Family Members

**Figure 1:**
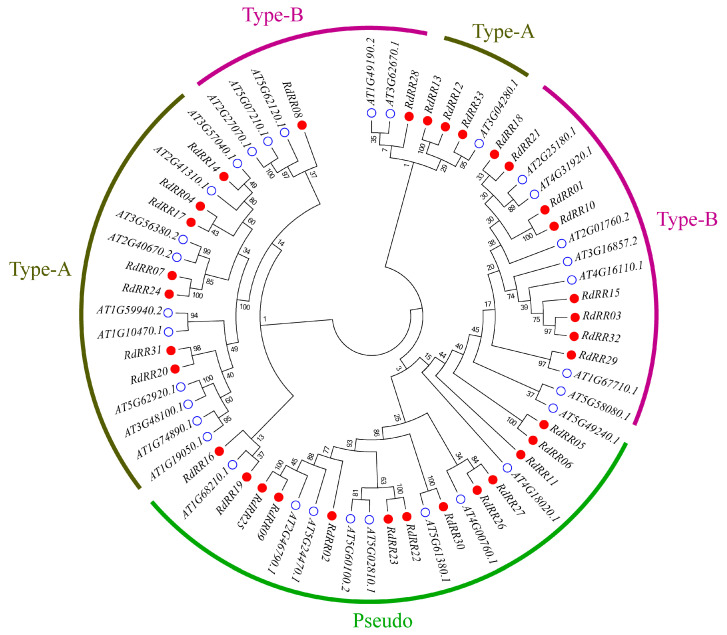
Phylogenetic tree analysis of RR family members from *Rhododendron delavayi* and *Arabidopsis*. The RRs were classified into three subfamilies. Different subfamilies are represented by different colors. The red dots are *R. delavayi*, and the white dots are *Arabidopsis*.

To assess the evolutionary relationships of *R. delavayi* RR proteins, we conducted a phylogenetic tree analysis of the 33 identified RdRR proteins and known *Arabidopsis thaliana* RR proteins. As seen in Figure 1, based on the classification of *Arabidopsis*, the RdRR proteins can be grouped into three types, namely Type-A, Type-B, and Pseudo; the first two types each have 10 family members, while the Pseudo type has 13 family members.

### 2.2. Analysis of Physical and Chemical Properties of R. delavayi RR Family Genes

The physicochemical properties and secondary structure of 33 *RdRR* family genes were analyzed (Table 1). These genes encoded a wide variety of amino acids, spanning from 128 aa (*RdRR31*) to 1096 aa (*RdRR27*) in length, with a molecular weight ranging from 14.43 to 123.56 kD. Further analysis revealed that the amino acid length encoded by the 10 RdRR proteins of Type-A varied from 128 aa (*RdRR31*) to 262 aa (*RdRR13*), whose molecular weight ranged from 14.43 to 29.80 kD. The RdRR proteins of Type-B and Pseudo varied considerably in amino acid length and molecular weight. Overall, their isoelectric point (pI) ranged from 4.85 (*RdRR08*) to 9.04 (*RdRR33*); among them, 28 proteins had an isoelectric point < 7 and were thus acidic proteins, and only five proteins (*RdRR27*, *RdRR25*, *RdRR12*, *RdRR13*, *RdRR33*) had an isoelectric point > 7 and were basic proteins. Their instability index ranged from 30.74 (*RdRR33*) to 87.52 (*RdRR31*), with 8 of the 33 RdRRs being stable proteins and the other 25 being unstable proteins. The aliphatic index spanned 60.03 (*RdRR23*) to 100.67 (*RdRR24*). The proteins’ hydrophilicity ranged from −0.945 (*RdRR23*) to −0.176 (*RdRR21*), indicating that all family members are hydrophilic proteins. Subcellular localization analysis revealed that the 33 RdRR family members located to five different sites: the nuclear, endoplasmic reticulum, cytoplasmic, mitochondrial, and plasma membrane. However, the majority (22 family members) localized to the nuclear, whereas only one family member localized to the plasma membrane.

By predicting their secondary structure, we found that all 33 family members consisted of an alpha helix, extended strand, beta turn, and random coil (Table 1). In general, both the alpha helix (18.34~54.37%) and random coil (25.42~64.79%) were dominant in every family member, while the beta turn (2.40~12.50%) was the least prevalent. After their detailed analysis, it was evident that the secondary structure of certain family members differed starkly. The highest percentage of random coil was found in 25 family members, of which 17 members had at least 50% of a random coil, whereas for the other 8 family members, a random coil reached more than 50% in one member. A total of four family members had a beta turn exceeding 10%, while two family members had less than 10% of the extended strand. Different structures also caused the RdRR proteins to engage in different functions.

### 2.3. Gene Structure and Conserved Motif Analysis of RdRR Family Members

**Figure 2:**
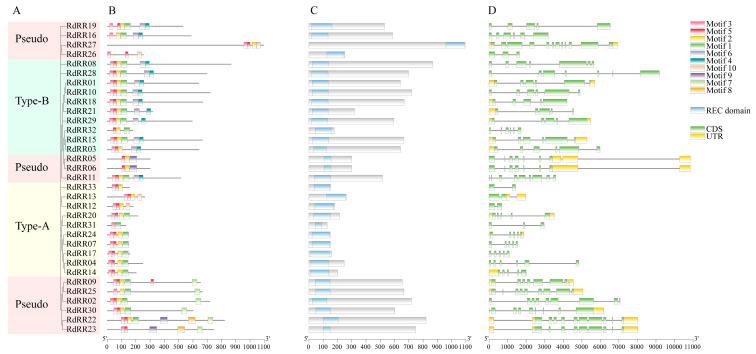
Evolutionary analysis, motif structure, conserved domain, and gene structure of *RdRRs*. (**A**) The phylogenetic tree of *RdRRs* was constructed by the NJ method with a 1000 bootstrap. (**B**) The motif structures were analyzed via the MEME database. Different motifs numbered 1–10 had different colors. (**C**) Conserved domain. Blue boxes indicate the REC domain. (**D**) Gene structure. Coding DNA sequence (CDS) regions and untranslated (UTRs) regions are represented by the green and yellow boxes, respectively.

To gain further understanding and information about the *RdRR* family of genes, a phylogenetic tree construction of their RdRR protein sequences was performed using MEGA (Figure 2A). These classification results agreed with those of the *Arabidopsis* analysis, in being divided into three types: Type-A, Type-B, and Pseudo. By analyzing the *RdRR* conserved domain of the gene (Figure 2C), we found that all 33 *RdRR* family genes harbored it (REC domain), further validating that all identified members indeed belonged to the *RR* gene family. A gene structure map was then derived (Figure 2D) to compare the intron, exon, and UTR composition of all *RdRR* genes according to gene annotations. These results showed that the *RdRR* family genes differed in their gene structure, having between 2 and 12 exons, and likewise between 2 and 12 introns. Among the 33 genes, *RdRR12*, *RdRR13*, and *RdRR33* had at least two exons, and all of them belonged to Type-A; in contrast, both *RdRR22* and *RdRR27* contained 10 exons, and these two genes belonged to the Pseudo type. A total of 11 genes had 5′ and 3′ terminal UTRs, of which five genes belonged to the Pseudo type. Overall, 7 of the 33 genes had one-terminal UTR, and *RdRR05* had three 3′ terminal UTRs.

**Figure 3:**
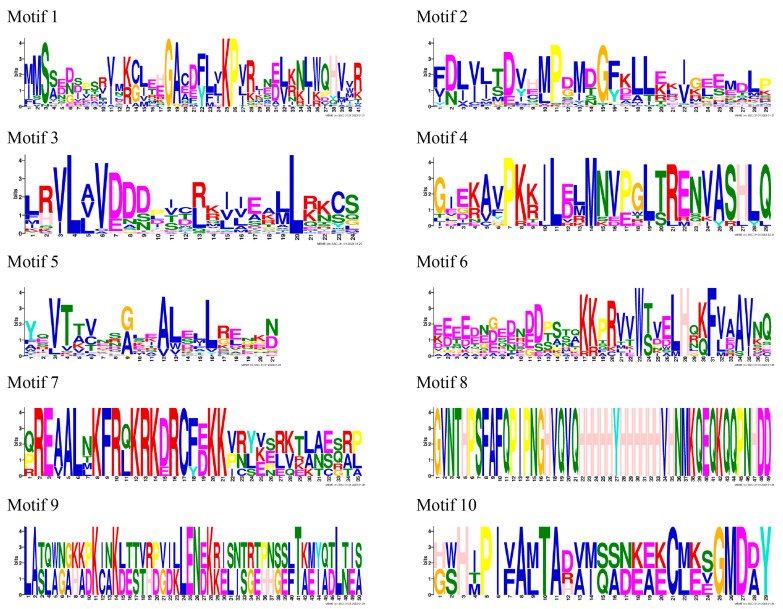
Structural characteristics of the *RdRR* gene family motif in *R. delavayi*. The abscissa refers to the amino acid with highest frequency, and the ordinate represents the relative frequency of the corresponding amino acid.

To further investigate the characteristic regions of the RdRR proteins, their motif structure diagram was constructed using the MEME database (Figure 2B), and the composition of each motif was analyzed (Figure 3). Ten motifs were identified in total; of them, motif 5 was present in all RdRR proteins except RdRR25 and RdRR31 and was the most conserved motif. Interestingly, RdRR09 contained two instances of motif 5, which consisted of 21 sites (YEVTTVNSGLEALELLRENKB). Motifs 2 and 3 were both identified in 30 RdRR proteins, with motif 2 consisting of 29 sites (FDLVJTDVHMPDMDGFKLLEKIGEEMDLP) and motif 3 consisting of 24 sites (LRVLAVDDDPICRKIIEALLRKCS). Motif 8, consisting of 49 sites (GVNTHPSFAFQPIPNGHVQVQQHHHHHHHHVHNMKQEQKQPNHDD), was identified in RdRR22 and RdRR23 only. Motifs 9 and 10 were identified exclusively in four RdRR family members. The number of identified motifs varied greatly among the 33 RdRR proteins, with a maximum of seven identified motifs for *RdRR22* and a minimum of two identified motifs (i.e., motif 1 and 3) for *RdRR31*. The motifs of RdRR proteins of different types were analyzed as well. Motif 3 was distinguishable in all 10 genes of Type-A but not motif 4, 6, 7, 8, or 9. Motif 5 was identified in all 10 genes of Type-B; however, these genes lacked motifs 7, 8, 9, and 10. One or more of the 13 genes in the Pseudo type were identified as motif 1–10, and motifs 7, 8, and 9 were restricted to the Pseudo type.

### 2.4. Chromosome Distribution of RdRR Family Genes

**Figure 4:**
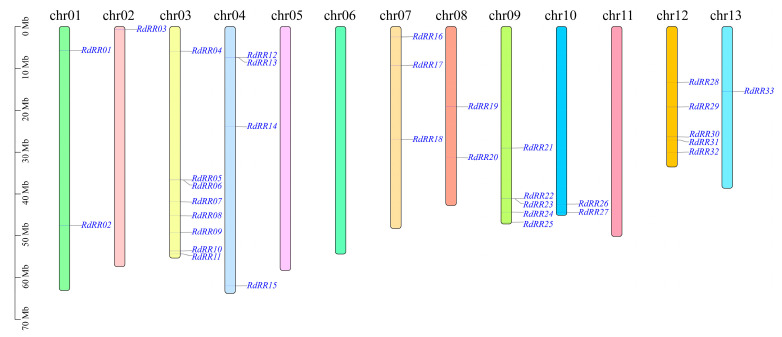
Chromosome location of the *RdRR* genes.

The distribution of *RdRR* family genes was determined by locating their positioning on the chromosome structure (Figure 4). These 33 *RdRR* genes were mapped to 10 (chr01, chr02, chr03, chr04, chr07, chr08, chr09, chr10, chr12, and chr13) of the 13 chromosomes in *R. delavayi*, with no *RdRR* genes found on chr05, chr06, or chr11. The most *RdRR* genes (*n* = 8) were mapped to chr03, while the fewest *RdRR* genes (*n* = 1) were mapped to chr02 and chr13. The analysis of *RdRR* gene replication events showed that no tandem events have occurred.

### 2.5. Gene Replication and Collinearity Analysis of RdRR Genes

**Figure 5:**
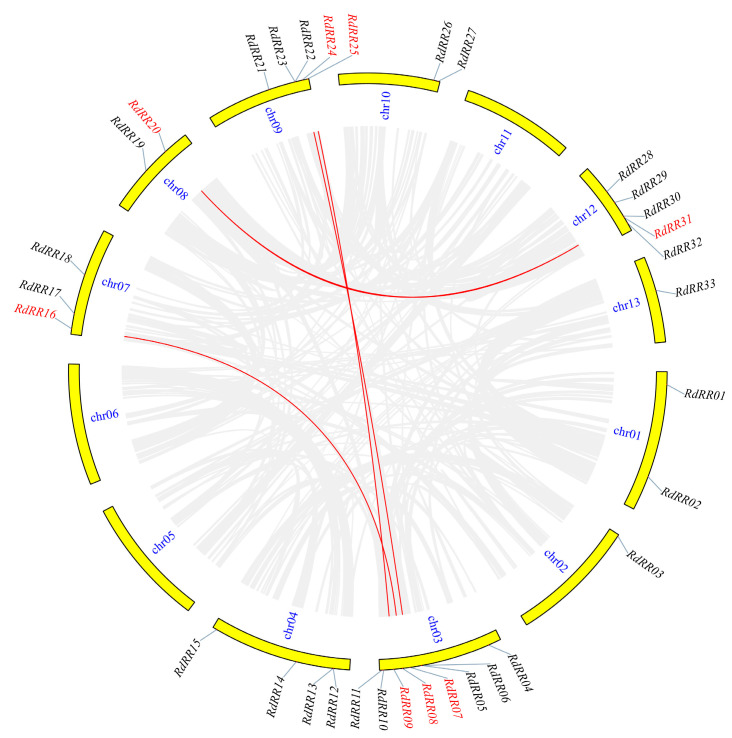
Synteny analysis of *RdRR* genes. The gray background represents synteny blocks within the whole genome; red fonts and lines indicate the syntenic *RdRR* gene pairs.

**Figure 6:**
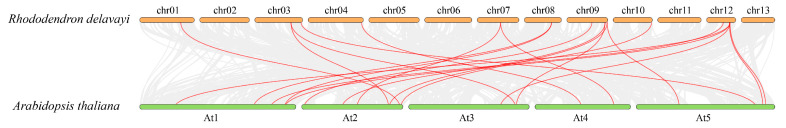
Collinearity analysis of *RdRR* genes with *Arabidopsis thaliana*. The gray background represents synteny blocks within the whole genome, and red lines represent the collinear gene pairs of *RR* genes.

By analyzing the gene replication events on different chromosomes (Figure 5), we detected four gene pairs of segmental replication events in *R. delavayi*, corresponding to *RdRR07*~*RdRR24*, *RdRR08*~*RdRR16*, *RdRR09*~*RdRR25*, and *RdRR20*~*RdRR31*. To better understand the origins of the *RdRR* gene family, we assessed the collinearity of *R. delavayi* and *Arabidopsis* across the genome (Figure 6). This revealed a total of 13 *RdRR* genes showing collinearity with *Arabidopsis* genes, for which 21 gene pairs coexisted. These genes therefore played an important role in the RR gene family’s expansion in *R. delavayi*.

### 2.6. Promoter Cis-Regulatory Element Analysis of the RdRR Genes

**Figure 7:**
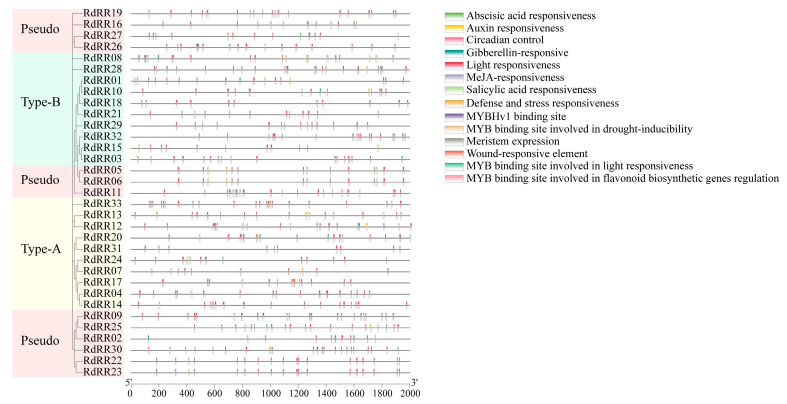
Promoter analysis of *RdRR* genes. Each motif is represented by a specific color.

The first 2.0 Kb *cis*-elements of the *RdRR* gene were studied using the Plant CARE database, and *cis*-elements of the promoter regions of each gene were identified. However, the distribution and quantity of these *cis*-regulatory elements varied greatly (Figure 7). All 33 genes harbored light response elements, including ACE, G-box, 3-AF1 binding site, AAAC-motif, AE-box, AT1-motif, ATC-motif, ATCT-motif, Box 4, and GATA-motif; however, their quantity per gene varied greatly. For example, we found more than 10 light-responsive elements in 20 *RdRR* genes, but the most (18) in *RdRR04*. Hormone-related response elements included MeJA-responsive elements (CGTCA-motif and TGACG-motif), gibberellin-responsive elements (P-box, GARE-motif, and TATC-box), auxin-responsive elements (TGA-element, AuxRR-core, TGA-box, AuxRE, and GATA-box), and abscisic acid- responsive elements (ABRE). Here, a total of 27 of the 33 genes were analyzed, and 101 abscisic acid-responsive elements were identified; their number peaked at 12 in *RdRR04*. A total of 28 gibberellin-responsive elements were identified from 19 genes, of which *RdRR02* had the most, totaling three. Some of the elements associated with plant stress responses were less frequent, with the wound-responsive element (WUN-motif) limited to *RdRR10*, and a total of 13 defense and stress response elements (TC-rich repeats) were identified from nine genes. A total of four *cis*-elements related to MYB binding sites were identified: MBS, MBSI, MRE, and CCAAT-box. Of these, 22 MBS *cis*-elements were identified in 16 genes, and three MBSI *cis*-elements were identified in two genes. *Cis*-elements associated with plant growth and development included meristem expression elements (CAT-box), as well as circadian control elements (circadian), the former being more common in the 33 genes studied here.

### 2.7. Expression Patterns of the RdRR Genes

**Figure 8:**
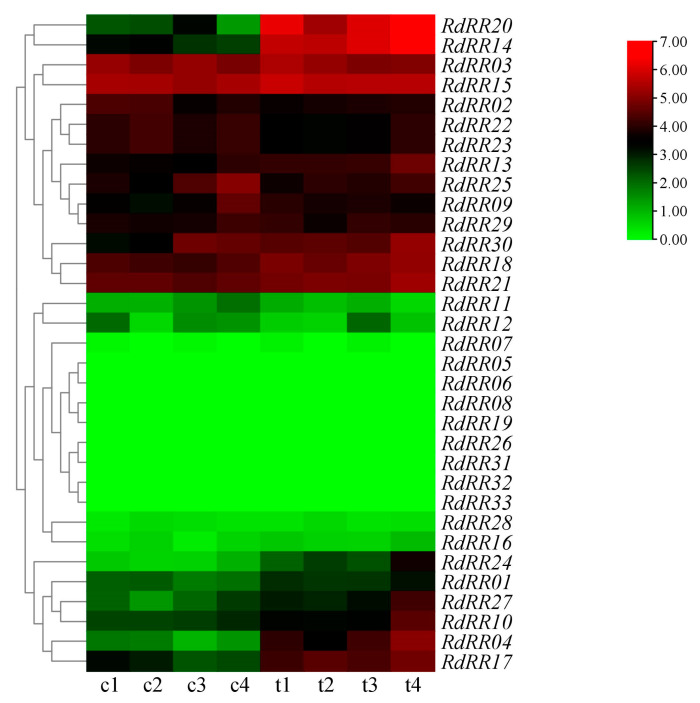
Expression pattern of *RdRR* genes in response to different treatments. c1–c4 and t1–t2 indicate the four developmental stages of adventitious buds’ formation from in vitro leaves under control (c) and treatment (t), respectively.

By analyzing the expression patterns of *RdRR* family genes in response to TDZ treatments (Figure 8), we uncovered a total of seven *RdRR* genes—*RdRR05*, *RdRR06*, *RdRR08*, *RdRR19*, *RdRR31*, *RdRR32*, and *RdRR33*—not expressed in the leaves of *R. delavayi*. Expression levels of *RdRR03*, *RdRR15*, and *RdRR21* were higher in the control group, showing small differences in expression levels through stages of growth and development. *RdRR07* was expressed exclusively in c1, c3, t1, and t3, but at very low levels, and this gene was not expressed in the other four treated groups. Expression levels of *RdRR16* and *RdRR28* were lower in every treatment. Compared with the control group, a total of 13 genes were expressed more after undergoing the TDZ treatment: *RdRR15*, *RdRR16*, *RdRR13*, *RdRR27*, *RdRR18*, *RdRR21*, *RdRR10*, *RdRR24*, *RdRR20*, *RdRR17*, *RdRR01*, *RdRR04*, and *RdRR14*. Among these, the expression level of *RdRR15* at the first and third developmental stages (i.e., t1 and t3) was significantly higher after the TDZ treatment than the control, and likewise for the expression levels of *RdRR04*, *RdRR17*, and *RdRR18* at the second and third developmental stages (i.e., t2 and t3). Similarly, the expression levels of *RdRR14* (Type-A) and *RdRR20* (Type-A) genes were lower in the control group but increased markedly after the TDZ treatment, being significantly higher than the control at all stages. Furthermore, after the TDZ treatment, the expression level of *RdRR20* was significantly lower at the second developmental stage (t2) of leaf differentiation than other stages. These results indicated that *RdRR14* and *RdRR20* could be important candidate genes induced by TDZ that can affect the differentiation and bud formation of leaves.

### 2.8. Morphological Differences of Three Rhododendron Genotypes Response to Exogenous TDZ

**Figure 9:**
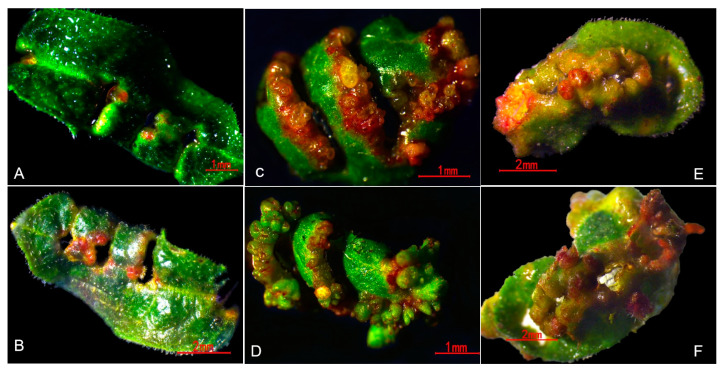
Differences in morphological response of three genotypes of Rhododendron in vitro leaves to exogenous TDZ (0.36 µM). (**A**,**B**), Re leaf culturing for 22 days and 28 days, respectively; (**C**,**D**), Rc leaf culturing for 15 days and 20 days, respectively; (**E**,**F**), Rd leaf culturing for 22 days and 28 days, respectively; Re denotes *Rhododendron* ‘Elisabeth Red’, Rs denotes *Rhododendron* ‘Scintillation’, and Rd denotes *Rhododendron delavayi*.

Under control treatment, the in vitro leaves of three genotypes of Rhododendrons did not differentiate into adventitious buds, while under TDZ treatment, the differentiation of adventitious buds among the three genotypes showed differences in time and morphology. After 15 days of inoculation, granular protuberance were formed at the incision site of Rs leaves (Figure 9C), and a large number of adventitious buds were formed after culturing 20 days (Figure 9D); whereas the incision became swelled without granular protuberance on Re leaves after culturing for 22 days (Figure 9A), and by 28 days, the swelling site turned brown or red, forming a very small number of adventitious buds (Figure 9B). After 22 days of inoculation, Rd’s leaf incisions swelled and formed a small number of granular protuberance (Figure 9E). Then, this protuberance developed into adventitious buds after 28 days (Figure 9F). This indicates that exogenous TDZ regulated the differentiation of adventitious buds from the in vitro leaves of Rhododendron, but different genotypes had different response effects.

### 2.9. Expression Analysis of Candidate RdRR Genes in Three Rhododendron Genotypes Differing in Their Regeneration Ability

**Figure 10:**
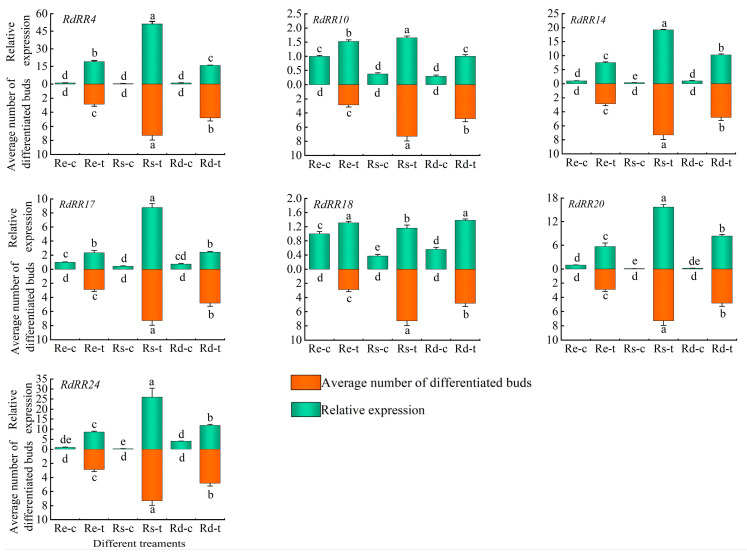
Number of adventitious buds and expression levels of candidate *RR* genes under the TDZ treatment in three *Rhododendron* genotypes. Note: Re denotes *Rhododendron* ‘Elisabeth Red’, Rs denotes *Rhododendron* ‘Scintillation’, and Rd denotes *Rhododendron delavayi*; ‘t’ refers to the treatment group, while ‘c’ is the corresponding control group; error bars represent the SD, and different letters indicate significant differences (*p* < 0.05, *n* = 3, one-way ANOVA, followed by Duncan’s test).

A total of seven candidates *RdRR* genes were selected from the transcriptome data for the qPCR analysis. As Figure 10 shows, no regenerated buds formed in the three genotypes of *Rhodendron* (i.e., one species and two cultivars) under the control treatment, whereas adventitious buds did form after the TDZ treatment. Yet there was a great difference in the number of adventitious buds among the three genotypes, being highest in Rs, followed by Rd, and lowest in Re, whose differences were significant (*p* < 0.05). By analyzing the expression levels of these seven candidates *RdRR* genes in the control and TDZ treatment groups of the three genotypes, those genes could be roughly divided into three classes. Expression levels of *RdRR10* and *RdRR18* increased after TDZ treatment, but the increase was small; the expression level of *RdRR10* was ranked Rs > Re > Rd, while that of *RdRR18* were Rd > Rs > Re. Hence, the expression trend for these two genes differed from the average number of adventitious buds after the TDZ treatment. Compared with the control group, the expression level of *RdRR4* in response to TDZ treatment was significantly increased (*p* < 0.05), and this increase was high, being significantly different among the three genotypes (*p* < 0.05). However, the expression level of *RdRR4* was ranked as Rs > Re > Rd, unlike the trend for the average number of adventitious buds after the TDZ treatment. Regarding the other four genes, *RdRR14*, *RdRR17*, *RdRR20*, and *RdRR24*, they formed the third group, given that their expression levels all significantly increased after the TDZ treatment vis-à-vis the control group, whose ranking was Rs > Rd > Re. This matched the trend found for the average number of adventitious buds after the TDZ treatment.

## 3. Discussion

The cytokinin RR, a key factor in cytokinin signaling, has been identified in several plant species. In a study of *Nicotiana tabacum*, 59 *NtRR* genes were identified, including 21 of Type-A and 38 of Type-B [18]. A total of 14 *JcRR* genes were identified in *Jatropha curcas*, consisting of 6 Type-A and 8 Type-B [19]. A total of 33 PtRR genes were identified in *Populus*, which were classified into three types: Type-A, Type-B, and Pseudo, with each type having 11 PtRR genes [20]. Likewise, the RR genes identified in *Arabidopsis* and rice (*Oryza sativa*) could also be classified into those three types. In this study of *R. delavayi*, 33 RR genes were distinguishable, comprising 10 family members each of Type-A and Type-B, with the other 13 being Pseudo. In comparison with other species, although the number of RR genes in *R. delavayi* has undergone expansion, it remains relatively lower. By analyzing gene replication events, we found that *RdRR07*~*RdRR24*, *RdRR08*~*RdRR16*, *RdRR09*~*RdRR25*, and *RdRR20*~*RdRR31* entailed segmental duplication events, which likely led to RR gene family expansion in this *Rhododendron*. In stark contrast, tandem duplication events in these RR genes were absent, perhaps contributing to the relatively small number of *RdRR* genes vis-à-vis other species. In general, the amplification of the RdRR family members may have arisen mainly via segmental duplication events, but most members of this family have no sign of amplification. This latter finding may be closely related to the loss of certain homologous genes in the process of chromosome duplication.

The RR family proteins in Type-A have a conserved L-aspartic acid, L-aspartic acid, lysine (D-D-K) region, and a short c-terminal whose function is unknown. The RR family proteins in Type-A are cytokinin-induced; thus, we speculate that following a cytokinin treatment the transcription of Type-A RR genes is accelerated. The RR gene of Type-B is a kind of plant-specific transcription factor, one having a long c-terminal sequence in addition to the D-D-K conservative sequence; the c-terminal’s long sequence contains the Myb-like DNA binding domain, known as the GARP region, which lies downstream of the target gene location of the cytokinin binding reaction [21,22]. The functional characteristics of RR family members are well studied [20,23,24,25,26], confirming that RR genes are crucial participants in the development and growth of different plants.

The RR gene of Type-B inhibits the activity of *ARR1*, providing an explanation for its negative feedback regulation of the cytokinin signal [27]. The expression of Type-B ARR affects the occurrence of the root apical meristem. During the root development of *Arabidopsis*, the formation and maintenance of its meristem are jointly regulated by cytokinin and auxin [28]. In *Populus*, the *PtRRI* (Type-A) transcription is induced by 6-BA, GA3, and IBA after treatment with these plant hormones. Unlike Type-A, transcription of the Type-B RR gene is not induced by cytokinin. In *Populus*, 6-BA, GA3, ABA, and IBA hormone treatments failed to alter the transcription level of *PtRR10/12d* (Type-B), which suggests the *PtRR10/12d* gene was not induced by phytohormones [29]. In *Arabidopsis*, the gene *ARR12* (Type-B) promotes regeneration yet *ARR1* inhibits regeneration in a manner dependent on *ARR12* [30]. *ARR1* inhibits axillary bud growth in *Arabidopsis* [31] whereas *SlRR10*, a homolog of *ARR1*, promotes axillary bud growth in tomatoes [32]. In rice, overexpressing *OsRR22* (Type-B) lines have smaller panicles and fewer branches [33], and overexpression of the *OsRR2* gene is known to decrease plant height [34]. In *Populus*, the *PtRR13* (Type-B) gene negatively regulates adventitious root development [29]. Due to the deletion of the L-aspartic acid site of the Pseudo protein, members of the Pseudo type mainly regulate the circadian rhythm of plants, but different members can function differently in this respect [35]. In the present study, TDZ was applied to *R. delavayi* leaves to induce bud differentiation, and their transcriptome was sequenced at four time points according to the morphological characteristics of adventitious bud formation. We found that seven *RdRR* genes were not expressed in leaves, while the other 26 genes were, though their expression levels differed significantly. Compared with the control group, 13 genes were expressed at higher levels after the TDZ treatment, of which seven genes—*RdRR04*, *RdRR17*, *RdRR18*, *RdRR10*, *RdRR14*, *RdRR20*, and *RdRR24*—showed significant trends of change among developmental stages or after the TDZ treatment. Based on the transcriptome results, further analysis (qPCR) of these seven *RdRR* genes as candidate differential genes revealed that they could be divided into two groups. By comparing the ability of bud regeneration of three *Rhododendron* genotypes (i.e., one species and two cultivars) when treated with TDZ, we found that expression levels of *RdRR04*, *RdRR10*, and *RdRR18* were inconsistent with the trend for the average number of differentiated buds. By contrast, those of *RdRR14*, *RdRR17*, *RdRR20*, and *RdRR24* were consistent with the changed average number of adventitious buds. Moreover, these latter four genes belonged to Type-A, in agreement with previous research reporting that Type-A RRs are induced by cytokinins.

In *Arabidopsis*, the function of the RR gene is well studied. For example, through the overexpression of *ARR7*, it was shown that this gene’s normal expression depends on cytokinin signaling, responding strongly to changes in cytokinin activity [36]. Another gene, *ARR9*, regulates the changes to the circadian rhythm, while *ARR5*, *ARR6*, *ARR7*, and *ARR15* together control meristem development, with *ARR7* and *ARR15* known to play important roles in the ecotype changes of root stem cells [37]. Investigating the overexpression of *Arabidopsis* Type-A response regulatory genes has also revealed their different roles and regulatory mechanisms in cytokinin signaling pathways [38]. Overexpressing the Type-A RR genes can result in multiple cytokinin-related phenotypes. The stability of *ARR16* and *ARR17* (possibly including *ARR8* and *ARR15*) is also regulated by cytokinins. The ARR protein is regulated by a combined mechanism entailing cytokinin and proteasome pathways, thereby playing a unique role in plant growth and development [22]. In the present study, the RR genes of *Arabidopsis* and *R. delavayi* were selected for an evolutionary tree analysis. The paramount candidate genes selected via the transcriptome and qPCR analyses all belonged to Type-A. Both *RdRR14* and *RdRR17* were most closely related phylogenetically to AT3G57040.1 (*ARR9*) and AT2G41310.1 (*ARR8*); likewise, the *RdRR20* was closest to AT1G19050.1 (*ARR7*), while *RdRR24* related most closely to both AT3G56380.2 (*ARR17*) and AT2G40670.2 (*ARR16*). When considered alongside previous *Arabidopsis* studies of the ARR gene, we can better predict the RR gene functioning in *R. delavayi* and verify the various functions of its members.

## 4. Materials and Methods

### 4.1. Identification of RR Family Genes in R. delavayi

The genome data for *R. delavayi* were downloaded from the Rhododendron Plant Genome Database (http://bioinfor.kib.ac.cn/rpgd/index.html, accessed on 31 January 2023). The *RdRR* family genes were identified via two methods. First, the local blast database was constructed, after which the RdRR protein sequences of related species from the National Center for Biotechnology Information (NCBI) database (https://www.ncbi.nlm.nih.gov/, accessed on 3 February 2023) were used for the local blast. Second, the hidden Markov model (PF00072) was downloaded from the Pfam (http://Pfam.xfam.org/, accessed on 3 February 2023) database, and hmmer 3.0 software was then used to construct a local protein database and conduct the search. For detected RR domains, the NCBI’s conserved domain database (https://www.ncbi.nlm.nih.gov/Structure/cdd/wrpsb.cgi, accessed on 6 February 2023) was used to map their conserved domain.

### 4.2. Analysis of Physical and Chemical Properties of R. delavayi’s RR Family Genes

The online database ProtParam (https://web.expasy.org/ProtParam/, accessed on 8 February 2023) was used to analyze the physical and chemical properties of *RdRR* family members. To predict their subcellular localization, we used the PSORT II Prediction database (https://PSORT.hgc.jp/form2.html, accessed on 8 February 2023), with their secondary structure analyzed using the SOMPA database (https://npsa-prabi.ibcp.fr/, accessed on 8 February 2023).

### 4.3. Analysis of Phylogenetic, Conserved Motifs, and Gene Structure of R. delavayi RR Family Members

*Arabidopsis* RR protein sequences were downloaded from the TAIR database (https://www.arabidopsis.org/, accessed on 27 October 2020). The identified RdRR and *Arabidopsis* RR protein sequences were multiple-sequence aligned in MEGA software (11.0), and their phylogenetic tree (bootstrap = 1000) was constructed by the Neighbor-Joining (NJ) method. Based on available genome annotation information, the conserved motifs of the RR family members of *R. delavayi* were analyzed via the MEME database (http://meme-suite.org/tools/meme, accessed on 31 January 2023). The motif value was set to 10, and the remaining parameters were set to default values. The corresponding intron–exon map was derived using TBtools software (V2.001).

### 4.4. Chromosomal Localization and Collinearity Analysis of R. delavayi RR Family Genes

The identified *RdRR* genes were mapped onto chromosomes according to the physical location information of the genome. Under its default parameters, MCScanX was used to analyze tandem and segmental duplication events in the whole genome of *R. delavayi*. Next, a genome-wide collinearity analysis of *R. delavayi* and *A. thaliana* was also performed using MCScanX (with default parameters) [39,40]. To visualize these findings, we used TBtools.

### 4.5. Promoter Analysis of R. delavayi RR Family Genes

The upstream 2.0-Kb DNA sequence of each *RdRR* gene was submitted to the PlantCARE database (http://bioinformatics.psb.ugent.be/webtools/plantcare/html/ accessed on 31 January 2023) to predict their cis-regulatory elements. For their visualization, TBtools was used.

### 4.6. Expression Analysis of R. delavayi RR Family Genes

Based on previous transcriptome data of in vitro leaves’ differentiation into adventitious buds as induced by exogenous TDZ [41] (these data have been deposited in the NCBI’s SRA database (accession number: SUB11178298), we analyzed the expression pattern of *RdRR* genes at four developmental stages of adventitious buds formation. These results were visualized with TBtools [42].

### 4.7. Response of In Vitro Leaves of Three Rhododendron Genotypes Induced by Exogenous TDZ

#### 4.7.1. Plant Materials and Treatments

Three *Rhododendron* genotypes (i.e., *Rhododendron* ‘Scintillation’ (Rs), *R. delavayi* (Rd), and *R*. ‘Elisabeth Red’ (Re)), whose regeneration ability differed, were selected as plant materials. Test-tube seedlings of these three genotypes of *Rhododendron* were cultured for 30 days in a medium consisting of WPM + ZT 2.27 µM + NAA 0.05 µM). After that, the first and second sets of functional leaves atop each plant were taken, and a wound cut was made to the surface of each leaf. Their leaf backsides were inoculated on adventitious bud induction medium (WPM is the basic medium added: 30 g/L sugar and 6.5 g/L agar). To the control (c) medium, only 0.05 µM NAA was added, while to the treatment (t), 0.36 µM TDZ and 0.05 µM NAA were added. Treatment and control groups were inoculated in 10 petri dishes, with 20 leaves per dish: 7 dishes were used for qRT-PCR, and the other 3 dishes for counting the average number of adventitious bud differentiated from the experimental leaves. All these materials were cultured under the same conditions: pH 5.4, 25 ± 2 °C, and 12 h/day of light (25 μmol·m^−1^·s^−1^).

#### 4.7.2. Response of Candidate RdRR Genes When Induced by Exogenous TDZ

We observed the morphological differences in the adventitious bud differentiation of in vitro leaves among these three Rhododendron genotypes. Granular protuberance formed at the incision site is a key morphological feature of leaf acquisition regeneration ability [38]; at this time, the expression levels of the candidate genes (selected in Section 4.6) were measured from the treated and control leaves. We calculated the average number of adventitious buds after ca. 50 days of inoculation (average number of adventitious buds = total number of adventitious buds with a length > 0.5 cm per treatment/number of inoculated leaves per treatment).

From each sample leaf, total RNA was extracted using a total RNA extraction kit (TSP412) (Tsingke Biotechnology Co., Ltd., Beijing, China). This extracted RNA was quantified by a Nanodrop spectrophotometer (Thermo Fisher Scientific Inc., Waltham, MA, USA), and its cDNA was synthesized using the Goldenstar RT6 cDNA Synthesis Mix (Tsingke Biotechnology Co., Ltd., Beijing, China). Quantitative real-time PCR (qPCR) was conducted using the ABI QuantStudioTM 1 Plus (Applied Biosystems, Thermo Fisher Scientific Inc., Waltham, MA, USA) and 2xT5 Fast qPCR Mix (SYBR Green I) (Tsingke Biotechnology Co., Ltd., Beijing, China). Each gene was examined using three replicates. The relative expression levels of each gene were calculated according to the 2^−ΔΔCt^ method. *Rd18S* served as the internal housekeeping gene. The primers for the qPCR can be found in Table S4.

## 5. Conclusions

Based on the genome data of *R. delavayi*, a total of 33 *RdRR* genes were identified. According to the classification of RR genes in *Arabidopsis*, these 33 *RdRR* genes could be divided into three types: Type-A, Type-B, and Pseudo. Analyzing the gene replication events uncovered no tandem duplications in these 33 *RdRR* genes, but eight genes did form segmental duplication events consisting of four gene pairs, which was likely a major reason of the family members’ expansion. Analysis of the *RdRR* gene based on the transcriptome data obtained after different treatments revealed that the expression levels of *RdRR10* (Type-B), *RdRR14* (Type-A), *RdRR20* (Type-A), and *RdRR24* (Type-A) in the control group were the lowest, but their expression levels were significantly higher under the TDZ treatment; further, the expression level of *RdRR20* was significantly lower at the second developmental stage (t2) of leaves’ differentiation than other stages in response to the TDZ treatment. For *RdRR14*, *RdRR17*, *RdRR20* and *RdRR24*, their expression levels were consistent with the average number of adventitious buds after the TDZ treatment; hence, we suggest those may be important candidate genes induced by TDZ. This study lays a solid foundation for the further analysis of cytokinin effects associated with the *RdRR* gene family.

## Figures and Tables

**Table 1:** Physicochemical properties and secondary structure of the *RR* gene family of *Rhododendron delavayi*.

Gene Name	Gene ID	Type	Number of Amino Acids (aa)	MW (kD)	pI	Instability Index	Aliphatic Index	GRAVY	PSORT II Prediction	Alpha Helix	Extended Strand	Beta Turn	Random Coil
*RdRR01*	Rhdel01G0042000.1	Type-B	641	69.91	6.18	52.73	69.50	−0.589	nuclear	19.97	10.61	4.68	64.74
*RdRR02*	Rhdel01G0241400.1	Pseudo	718	79.53	5.70	50.62	71.85	−0.498	nuclear	23.82	10.17	2.79	63.23
*RdRR03*	Rhdel02G0007300.1	Type-B	642	71.55	6.75	51.06	82.73	−0.488	nuclear	26.64	11.68	4.83	56.85
*RdRR04*	Rhdel03G0038500.1	Type-A	248	28.30	4.91	57.45	82.50	−0.697	nuclear	25.00	14.52	5.24	55.24
*RdRR05*	Rhdel03G0190600.1	Pseudo	300	33.73	6.41	56.28	87.00	−0.355	nuclear	23.67	22.67	10.67	43.00
*RdRR06*	Rhdel03G0190600.3	Pseudo	300	33.73	6.41	56.28	87.00	−0.355	nuclear	23.67	22.67	10.67	43.00
*RdRR07*	Rhdel03G0224400.1	Type-A	152	16.86	6.59	44.00	85.26	−0.374	nuclear	40.79	14.47	12.50	32.24
*RdRR08*	Rhdel03G0253000.1	Type-B	867	94.59	4.85	43.14	82.70	−0.318	endoplasmic reticulum	25.03	16.84	6.00	52.13
*RdRR09*	Rhdel03G0286200.1	Pseudo	654	72.84	6.18	49.85	67.06	−0.717	nuclear	29.82	8.10	3.06	59.02
*RdRR10*	Rhdel03G0329300.1	Type-B	721	80.08	5.59	42.13	79.72	−0.481	nuclear	23.30	11.93	4.16	60.61
*RdRR11*	Rhdel03G0337700.1	Pseudo	516	58.19	6.59	40.08	75.39	−0.508	cytoplasmic	26.74	11.82	5.43	56.01
*RdRR12*	Rhdel04G0061400.1	Type-A	181	20.47	8.34	55.53	82.43	−0.276	mitochondrial	48.62	9.94	7.73	33.70
*RdRR13*	Rhdel04G0061700.3	Type-A	262	29.80	8.55	57.18	88.17	−0.227	cytoplasmic	43.51	14.89	8.40	33.21
*RdRR14*	Rhdel04G0164500.1	Type-A	202	22.87	5.67	56.46	77.13	−0.704	nuclear	29.70	15.84	6.93	47.52
*RdRR15*	Rhdel04G0379000.1	Type-B	666	73.42	5.83	48.75	74.01	−0.524	nuclear	23.87	9.01	5.26	61.86
*RdRR16*	Rhdel07G0022900.1	Pseudo	587	65.44	6.17	41.24	73.27	−0.520	nuclear	33.73	19.08	8.86	38.33
*RdRR17*	Rhdel07G0078800.1	Type-A	159	17.88	6.29	47.56	90.06	−0.389	nuclear	28.93	18.87	8.81	43.40
*RdRR18*	Rhdel07G0201100.1	Type-B	668	73.20	6.68	35.84	75.28	−0.509	nuclear	19.76	10.93	4.94	64.37
*RdRR19*	Rhdel08G0149200.1	Pseudo	530	58.88	6.09	32.24	69.75	−0.548	nuclear	30.38	12.83	4.53	52.26
*RdRR20*	Rhdel08G0211800.1	Type-A	215	23.98	6.90	62.44	82.88	−0.499	nuclear	26.05	12.56	7.44	53.95
*RdRR21*	Rhdel09G0148100.1	Type-B	320	36.31	5.39	47.86	99.50	−0.176	endoplasmic reticulum	42.19	12.19	4.06	41.56
*RdRR22*	Rhdel09G0233700.1	Pseudo	821	89.92	6.74	38.56	64.69	−0.836	nuclear	22.05	10.84	3.41	63.70
*RdRR23*	Rhdel09G0233700.2	Pseudo	747	81.51	6.30	37.48	60.03	−0.945	nuclear	18.34	13.52	3.35	64.79
*RdRR24*	Rhdel09G0262600.1	Type-A	150	16.69	5.90	30.84	100.67	−0.239	cytoplasmic	35.33	16.00	4.67	44.00
*RdRR25*	Rhdel09G0287600.1	Pseudo	666	73.38	8.25	45.77	65.60	−0.677	nuclear	32.43	7.36	2.40	57.81
*RdRR26*	Rhdel10G0266900.1	Pseudo	252	28.59	6.14	42.98	85.16	−0.270	endoplasmic reticulum	54.37	11.90	6.35	27.38
*RdRR27*	Rhdel10G0282900.1	Pseudo	1096	123.56	7.90	38.66	91.33	−0.233	plasma membrane	41.70	13.96	4.56	39.78
*RdRR28*	Rhdel12G0100600.1	Type-B	698	77.49	6.38	46.32	71.05	−0.599	nuclear	32.23	15.04	6.45	46.28
*RdRR29*	Rhdel12G0133700.1	Type-B	595	67.32	5.85	40.68	75.66	−0.502	nuclear	21.18	13.78	3.53	61.51
*RdRR30*	Rhdel12G0195700.1	Pseudo	602	67.59	5.59	50.05	63.62	−0.674	nuclear	22.76	13.95	5.15	58.14
*RdRR31*	Rhdel12G0203900.1	Type-A	128	14.43	5.65	87.52	79.22	−0.855	mitochondrial	27.34	15.63	7.81	49.22
*RdRR32*	Rhdel12G0234000.1	Type-B	177	20.39	5.95	37.97	92.99	−0.216	cytoplasmic	49.15	20.34	5.08	25.42
*RdRR33*	Rhdel13G0133600.1	Type-A	154	17.00	9.04	30.74	70.84	−0.297	mitochondrial	36.36	21.43	10.39	31.82

## Data Availability

Data are available in the manuscript and in the Supplementary Materials.

## Supplementary Materials

The following supporting information can be downloaded at: https://www.mdpi.com/article/10.3390/plants12183250/s1, Table S1: RR protein sequences of *Rhododenron delavayi* and *Arabidopsis thaliana* were used to construct the phylogenetic tree, Table S2: Putative cis-acting elements identified in the promoter regions of RdRRs, Table S3: The expression levels (FPKM value) of RdRR genes in 4 developmental stages of buds formation from in vitro leaves of *Rhododendron delavyi* induced by TDZ, Table S4: Primers for qPCR.
